# Measurement properties of PROMIS short forms for pain and function in patients receiving knee arthroplasty

**DOI:** 10.1186/s41687-023-00559-x

**Published:** 2023-02-28

**Authors:** Anika Stephan, Vincent A. Stadelmann, Stefan Preiss, Franco M. Impellizzeri

**Affiliations:** 1 Department of Teaching, Research and Development – Lower Extremities, Schulthess Clinic, Lengghalde 2, 8008 Zurich, Switzerland; 2 Knee Surgery, Schulthess Clinic, Lengghalde 2, 8008 Zurich, Switzerland; 3 Faculty of Health, University of Technology Sydney, PO Box 123, Broadway, NSW 2007 Australia

**Keywords:** PROMIS, Short forms, Psychometric validation, Pain, Function, Knee arthroplasty, Responsiveness, Minimal important change

## Abstract

**Background:**

While there are a few studies on measurement properties of PROMIS short forms for pain and function in patients with knee osteoarthritis, nothing is known about the measurement properties in patients with knee arthroplasty. Therefore, this study examined the measurement properties of the German Patient-Reported Outcomes Measurement Information System (PROMIS) short forms for pain intensity (PAIN), pain interference (PI) and physical function (PF) in knee arthroplasty patients.

**Methods:**

Short forms were collected from consecutive patients of our clinic's knee arthroplasty registry before and 12 months post-surgery. Oxford Knee Score (OKS) was the reference measure. A subsample completed the short forms twice to test reliability. Construct validity and responsiveness were assessed using scale-specific hypothesis testing. For reliability, Cronbach’s alpha, intraclass correlation coefficients, and agreement using standard error of measurement (SEM_agr_) were used. Agreement was used to determine standardised effect sizes and smallest detectable changes (SDC90). Individual-level minimal important change (MIC) was calculated using a method of adjusted prediction.

**Results:**

Of 213 eligible patients, 155 received questionnaires, 143 returned baseline questionnaires and 119, 12-month questionnaires. Correlations of short forms with OKS were large (│r│ ≥ 0.7) with slightly lower values for PAIN, and specifically for men. Cronbach’s alpha values were ≥ 0.84 and intraclass correlation coefficients ≥ 0.90. SEM_agr_ were around 3.5 for PAIN and PI and 1.7 for PF. SDC90 were around 8 for PAIN and PI and 4 for PF. Follow-up showed a relevant ceiling effect for PF. Correlations with OKS change scores of around 0.5 to 0.6 were moderate. Adjusted MICs were 7.2 for PAIN, 3.5 for PI and 5.7 for PF.

**Conclusion:**

Our results partly support the use of the investigated short forms for knee arthroplasty patients. The ability of PF to differentiate between patients with high perceived recovery is limited. Therefore, the advantages and disadvantages should be strongly considered within the context of the intended use.

**Supplementary Information:**

The online version contains supplementary material available at 10.1186/s41687-023-00559-x.

## Introduction

The Patient Reported Outcomes Measurement Information System (PROMIS®) is a common health metric for many medical conditions primarily designed for computer adaptive testing (CAT) [1]. Several research projects including patients with knee arthroplasty have recently applied PROMIS CAT [2–5]. Nevertheless, PROMIS static short forms remain in use for the principal reasons that CAT may be unavailable in the target language or there is a lack of technical resources. Certain patient groups, particularly older adults, also still prefer paper-based surveys as observed in a recent health survey in Switzerland [6], where 24% of the youngest participants (15–24 years) and up to 80% of the oldest (75 years and older) chose paper. In these circumstances, the shortest PROMIS forms (≤ four items) are practical options for undertaking routine clinical evaluations. Their brevity minimises respondent and administrative burden, both potential barriers to the collection of patient-reported measures for registry documentation.

Pain and function are two relevant constructs for patients receiving knee arthroplasty. While there are a few studies on measurement properties of PROMIS short forms for pain and function in patients with knee osteoarthritis [7–9], nothing is known about the measurement properties in patients undergoing knee arthroplasty even though these tools are regarded as potentially useful for decision making [10] and have already been used for research in this patient group [11, 12]. Two validation studies including osteoarthritis patients applied short forms of 6-item (pain interference) or 10-item (physical function) length [7, 8], yet work examining the measurement properties of such or even shorter forms are still lacking. Of note, the 4-item static short forms for pain interference and physical function are part of the PROMIS-29 profile and “Impact Stratification Score” that were originally proposed for chronic low back pain [13], but might also be useful for total knee arthroplasty patients.

Therefore, the aim of the study was to determine the psychometric characteristics of the German PROMIS short forms for pain intensity (PAIN), pain interference (PI) and physical function (PF) in patients with knee arthroplasty. Specifically, we evaluated construct validity, internal consistency, test–retest reliability, responsiveness, floor and ceiling effects, and calculated the individual-level minimal important change for each scale. We used the COnsensus-based Standards for the selection of health status Measurement INstruments (COSMIN) as a guiding framework for conducting our analyses, defining thresholds, sample sizes and reporting [14, 15].

## Materials and methods

### Study design and questionnaire administration

**Fig. 1:**
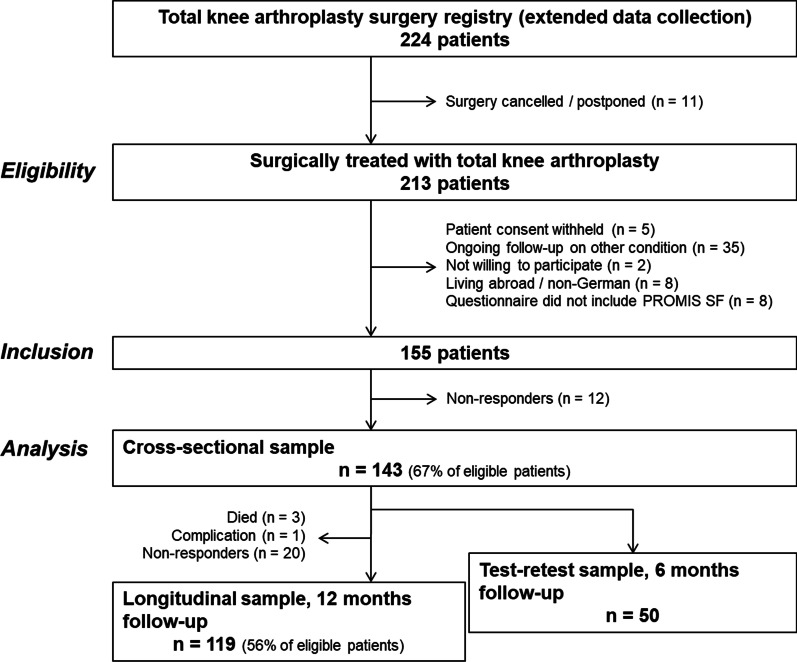
Flow chart showing patient eligibility and sample sizes for assessing German PROMIS short form measurement properties

This prospective study was approved by the Cantonal Ethics Committee of Zurich (KEK-ZH no. 2015-0258). We included consecutive patients from our knee arthroplasty registry who had undergone surgery within two cycles of extended data collection (November to December 2016 and July 2017) and received the PROMIS short forms in addition to their standard set of patient-reported outcome questionnaires (Fig. 1). Based on COSMIN guidelines for longitudinal and construct validity [16], we aimed for a sample size of at least 100, which is considered adequate for correlational analysis.

Enrolled patients had to provide consent to use their data for research purposes. The exclusion criteria included living abroad, insufficient knowledge of the German language, cognitive impairment or ongoing follow-up of knee arthroplasties at the other leg. These exclusion criteria are tied to the registry and aim to reduce the response burden (i.e., receiving too many questionnaires to complete) for the individual patient. Patient-reported outcomes were collected from paper questionnaires or digital versions administered 1 to 4 weeks before surgery (baseline) and at the 12-month follow-up. For reliability testing, a subsample of consecutive patients addressed for their baseline or 6-month follow-up registry survey completed questionnaires with a retest occurring within 2 to 14 days. The condition of patients before surgery was considered as stable. Also, the condition of patients 6 months after surgery tends to remain stable, as most change occurs within 3 months post-surgery [17]. For our purposes, we chose a sample size of 50, which is the suggested minimum for reliability testing [18].

### Outcome questionnaires

We investigated PROMIS short forms for PAIN (3 items), PI and PF (each with 4 items) provided by the PROMIS Germany research group. Answers are given on 5-point verbal rating scales. For PAIN, we used the form 3a (v2.0) that assesses pain over a 7-day recall period and current pain [19]. Form 4a (v1.0) defined PI based on the consequences of pain on relevant aspects of one’s life over a 7-day recall period [20, 21]. For PF, we used form 4a (v2.0) [22, 23] assessing the current ability to perform various physical activities. Overall scores for PAIN, PI and PF were presented as T-scores; higher scores indicate more PAIN, higher PI, and better PF. A score of 50 (10) represents the US general population mean (standard deviation). Scoring was done by using the “HealthMeasures Scoring Service”, powered by Assessment Center^SM^ (https://www.assessmentcenter.net/ac_scoringservice). Missing items were not replaced.

The reference measure used for this study was the Oxford Knee Score (OKS), a condition-specific instrument that assesses constructs encompassing the selected PROMIS domains. Specifically, we used the cross-culturally adapted and validated German OKS [24], which is a reliable and responsive 12-item, joint-specific self-administered questionnaire for assessing pain and disability in patients with knee arthroplasty. Items are answered on 5-point Likert scales extending from 0 to 4 points, where 4 indicates the best outcome. Total scores, calculated by adding all items, range from 0 (worst) to 48 points (best).

Additionally, patients rated their global treatment outcome (GTO) at 12 months using a single-item transition question to rate their health change after surgery [25]: “*How much did the operation help your knee problem?*” on a 5-point Likert scale ranging from “helped a lot” to “made things worse”. This global rating scale (transition item) was developed at our clinic and is used as an external criterion for treatment outcome. The construct validity of this global outcome scale was shown and discussed in a study on back pain patients [26]. Its reliability was investigated in patients undergoing surgery for lumbar spinal stenosis and resulted in an ICC(2,1) of 0.75 and a Kappa value of 0.73 [27], which can be interpreted as "acceptable" or "good" [28]. For the reliability estimate of our TKA population, we conducted a confirmatory factor analysis [29] using all available baseline and 6-month follow-up OKS scores in our clinic's knee arthroplasty registry (n = 4661). We found the R^2^ to be 0.70 and used this value for further calculations. Since pain and function deficit are among the main reasons for undergoing TKA surgery, it is reasonable to assume that for the surgery to help (or to help a lot), improvements in pain and function must be the main driver for the transition rating.

### Evaluation of measurement properties

Construct validity was assessed using scale-specific hypothesis testing. This involved examining correlations with the OKS total score at baseline and 12 months, each for the total sample and by gender. Therefore, there were a total of six hypotheses per scale and the test was considered good if at least 75% of the hypotheses were confirmed [28]. We used the Spearman rank correlation (r_s_) when non-normal distributions (Shapiro–Wilk test) were involved and the Pearson’s correlation coefficient (r) in all other cases. All correlations were expected to be large (confidence intervals ≥ 0.5). The correlations were expected to be negative for PAIN and PI with OKS, and positive for PF with OKS.

Internal consistency was calculated using Cronbach’s alpha with values between 0.70 and 0.95 indicating appropriate internal consistency [18]. The test–retest sample comprised 14 patients measured at baseline and 36 patients measured at 6 months follow-up. Since the examined PROMs can be used both before and after surgery, combining patients from both time points was a reasonable approach. The median test–retest response interval was 6 days. The mean score difference between test and retest was smaller than 1.2 T-score points in all three PROMIS scales (*p* > 0.2) with the 95% confidence interval including zero, which suggests a stable condition. Test–retest reliability was assessed with the intraclass correlation coefficient (ICC) from a single measurement, absolute agreement, 2-way mixed-effects model; an ICC (confidence interval) ≥ 0.7 was considered acceptable [18]. Agreement was assessed using the standard error of measurement (SEM_agr_) = √(variance due to systematic differences between measurements + residual variance) [28]. The effect size based on SEM_agr_ was calculated from the mean change score. The smallest detectable change (SDC) for individuals that can be considered above the measurement error with a 90% confidence level was calculated as SDC90 = 1.65 * √2 * SEM_agr_ [28].

Responsiveness defines the ability of a questionnaire to inform about clinically important changes over time. Longitudinal validity can be considered a measure of responsiveness and is examined by inspecting the correlation between change scores of the instrument under validation and the reference instrument. Change scores were calculated by subtracting baseline from follow-up scores. Considering the direction of each scale, negative change scores of PAIN and PI and positive change scores of PF and OKS correspond to an improvement in pain and function. We expected negative correlations between change scores of PAIN, PI and OKS, and positive correlations between change scores of PF and OKS, each in the order of |r| (confidence intervals) ≥ 0.5. The smallest effect size of interest was defined by Cohen’s *d* ≥ 1.5 for the decrease in PAIN and PI and the increase in PF. We yielded this threshold using PF and PI CAT results from a recovery curve of patients after total knee arthroplasty (TKA) [4] and a standard deviation of 5. Overall, we tested the correlation of change scores and the amount of effect size, each for the total sample and by gender, which constituted six hypotheses per scale. Responsiveness was considered sufficient if at least 75% of the hypotheses were confirmed [28].

Floor and ceiling effects were considered acceptable if percentages were below 15% [30]. Because of the different directions between scales, we defined ceiling effects as the score that indicates the best possible state, whereas floor effects apply to the score that indicates the worst possible state.

The minimal important change (MIC) that can be applied to the average TKA patient was calculated for each PROMIS scale from the receiver operating characteristic (ROC) curve (MIC_ROC_) as well as the predicted MIC (MIC_pred_) and adjusted predicted MIC (MIC_adj_) following the procedure of Terluin et al. [31]. MIC_ROC_ is defined as the change score cut-off to optimally classify improved and non-improved patients and was shown to be biased when the proportion of improved patients deviates from 0.5 [32, 33]. MIC_pred_ calculates the score change that is equally likely to occur in improved and non-improved patients and is also biased by group proportion [31]. MIC_adj_ considers the proportion of improved patients, the reliability of the transition rating, the correlation of the change score with the dichotomized transition rating and the standard deviation of the change score. As a precondition for MIC analysis, the Spearman rank correlation (r_s_) between the GTO and change score should be larger than 0.3 [34]. This threshold might seem low, but one must consider that while transition scores correlate with pain, disability and quality of life measures, they do include additional information about what the patient considers important in their individual clinical context [35]. Patients who stated that the operation “helped” or “helped a lot” were considered as having a good outcome; all other responses including "helped only little" indicated a poor outcome.

All analyses were performed using Stata Statistical Software Release 17 (StataCorp LP, TX, USA) as well as R version 4.2.2 [36] and the "lavaan" package [37].

## Results

Table 1 presents the baseline demographics with pain and function status. The age range was 48 to 88 years (median: 69). While women were mostly aged around 65 to 75 years, men had a flatter age distribution. Most surgeries were primary total arthroplasties (78%) with the remaining interventions including primary partial arthroplasties (10%) and arthroplasty revisions (13%). The baseline T-scores of PAIN and PI were considerably larger than in the reference population and PF was considerably lower; these scores normalised 12 months after surgery.

**Table 1:** Baseline patient characteristics and score changes

Characteristics^a^	Baseline (N = 143)	Longitudinal (N = 119)	Test–retest (N = 50)
Age (years)	68.3 (8.9)	68.5 (9.0)	69.3 (8.5)
Gender (men, women) (n, %)	57, 86 (39.9, 60.1)	49, 70 (41.2, 58.8)	14, 36 (28.0, 72.0)
Height (cm)	169.1 (9.7)	169.6 (9.7)	166.0 (8.4)
Weight (kg)	79.9 (16.9)	79.3 (17.0)	76.2 (14.8)
Body mass index (kg/m^2^)	27.8 (4.7)	27.4 (4.6)	27.6 (4.4)
PROMIS PAIN (T-score)	65.2 (7.7)	64.5 (7.7)^b^	65.6 (6.6)
PROMIS PI (T-score)	64.8 (5.8)	64.4 (5.9)	66.1 (5.6)
PROMIS PF (T-score)	36.6 (4.9)	36.9 (5.0)^b^	35.2 (4.5)
OKS	23.8 (8.4)^c^	24.3 (8.5)^c^	22.1 (7.3)
PROMIS PAIN (T-score change, 95% CI)		− 19.6 (− 21.5 to − 17.6)	
PROMIS PI (T-score change, 95% CI)		− 16.1 (− 17.6 to − 14.7)	
PROMIS PF (T-score change, 95% CI)		10.9 (9.8 to 12.0)	
OKS (score change, 95% CI)		16.9 (15.5 to 18.3)	

*PROMIS* Patient Reported Outcomes Measurement Information System; *PAIN* pain intensity; *PI* pain interference; *PF* physical function; *T-score* overall PROMIS score calculated per domain; *OKS* Oxford Knee Score; *CI* confidence interval

^a^Expressed as mean with standard deviation, unless otherwise stated

^b^For two cases, all short form items were missing and scores could not be calculated

^c^For one case, one item was missing and replaced by the mean of all other items to calculate the score

### Construct validity

Scale-specific hypothesis testing resulted in four of six (75%) confirmed hypotheses for PAIN and all six (100%) confirmed hypotheses for PI and PF (Table 2).

**Table 2:**
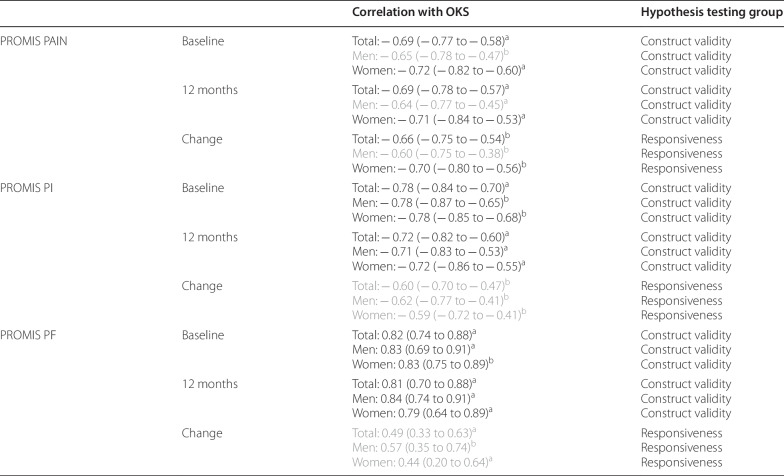
Correlations between PROMIS scales and the OKS

*PROMIS* Patient Reported Outcomes Measurement Information System; *OKS* Oxford Knee Score; *PAIN* pain intensity; *PI* pain interference; *PF* physical function; black versus grey font color: the confidence interval of the correlation does not overlap/overlaps with the preset correlation threshold

^a^Spearman's rank correlation coefficient, r_s_

^b^Pearson’s correlation coefficient, r

### Reliability

Cronbach’s alpha ranged between 0.84 and 0.90 (Table 3). The lower limits of all ICC confidence intervals were greater than 0.7. For each of the short forms, 18% to 20% of the test–retest sample had the best possible scores on both test occasions. Both the SEM_agr_ and SDC90 were higher for PAIN and PI compared to PF. The effect size based on SEM_agr_ was around 5 to 6 for all three short forms, and smaller than that for the OKS (9.5).

**Table 3:** Reliability, agreement and smallest detectable change

	Cronbach’s α^a^	ICC^a^	SEM_agr_	SDC90	Effect size based on SEM_agr_
PROMIS PAIN	0.84 (0.79 to 0.88)	0.93 (0.88 to 0.96)	3.55	8.28	5.51
PROMIS PI	0.90 (0.87 to 0.92)	0.90 (0.83 to 0.94)	3.34	7.78	4.84
PROMIS PF	0.88 (0.84 to 0.91)	0.97 (0.94 to 0.98)	1.72	4.02	6.33
OKS	–	–	1.78	4.15^b^	9.52

*ICC* intraclass correlation coefficient; *SEM*_agr_ agreement for T-scores assessed using standard error of measurement from test–retest; *SDC90* smallest detectable change for individuals that can be considered above the measurement error with a 90% confidence level;* Effect size based on SEM, *_agr_ calculated as absolute value of the mean change score divided by SEM_agr_ *PROMIS* Patient Reported Outcomes Measurement Information System; *PAIN* pain intensity; *PI* pain interference; *PF* physical function; *OKS* Oxford Knee Score

^a^95% confidence interval in parentheses

^b^According to: Beard DJ, Harris K, Dawson J, Doll H, Murray DW, Carr AJ, et al. Meaningful changes for the Oxford hip and knee scores after joint replacement surgery. J Clin Epidemiol. 2015;68(1):73–9

### Responsiveness

**Fig. 2:**
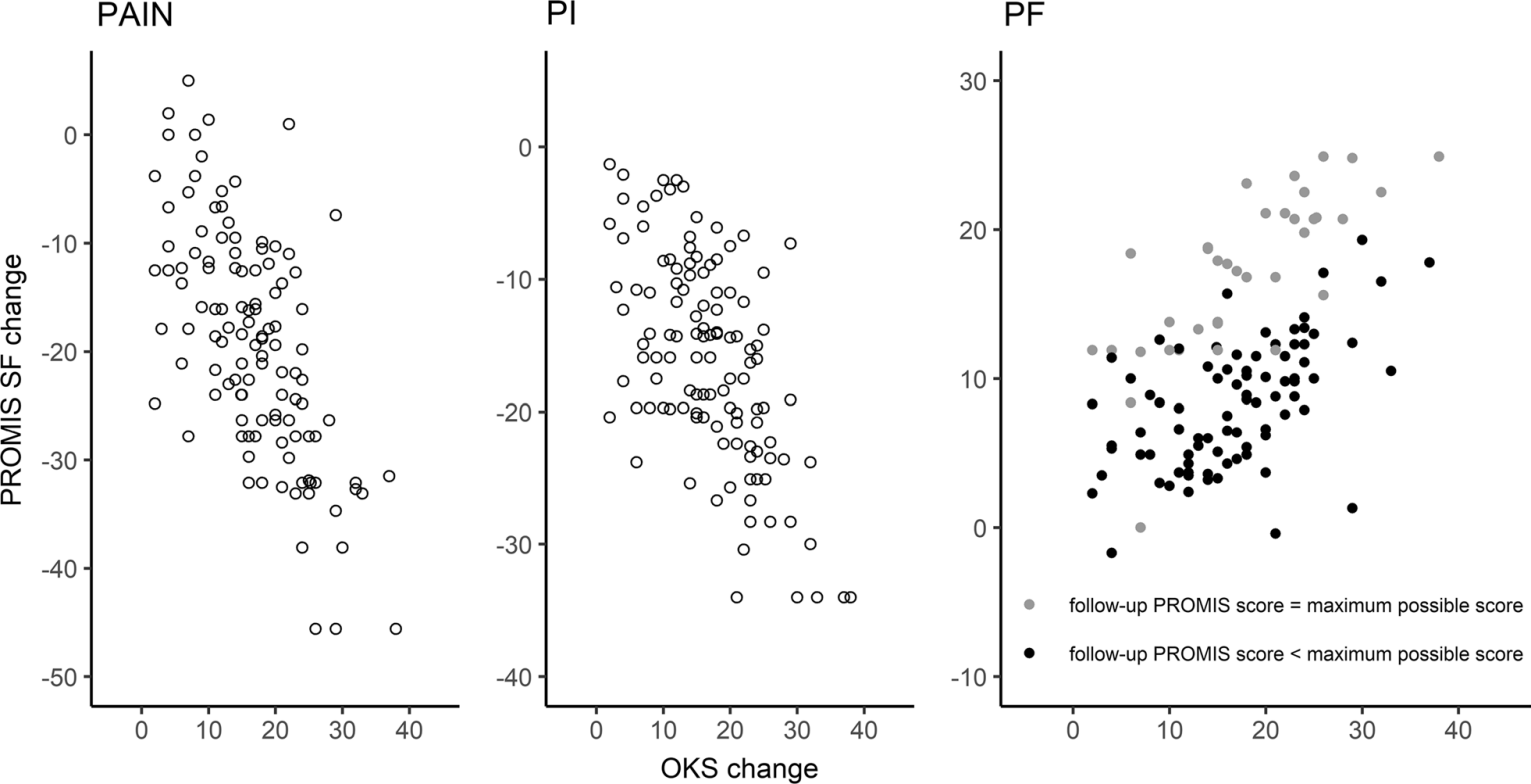
Responsiveness plots for PAIN, PI and PF with the latter highlighting if the patient achieved the best possible score or not

The confidence intervals |r| for the correlation of change scores overlapped the threshold of 0.5 for PI and PF, and for PAIN in men only (Table 2). The correlation plots are presented in Fig. 2.

Cohen’s *d* (95% confidence interval) values were 2.3 (1.9 to 2.8) for PAIN, 2.3 (1.9 to 2.7) for PI, and 1.7 (1.4 to 1.9) for PF. For the subsamples of women and men, Cohen’s *d* was in the range of 1.6 to 3.2. Thus, all hypotheses on effect sizes were confirmed. Overall, hypothesis testing for responsiveness resulted in five of six (83%) confirmed hypotheses for PAIN, and three of six (50%) confirmed hypotheses for PI and PF.

At baseline, the worst possible score for PAIN was recorded in 5.6% of the patients. The respective percentages are 10.5% for PI and 0.7% for PF. One patient achieved the best possible score for PF, whereas there were no such cases at baseline for PAIN and PI. At follow-up, the best possible scores for PAIN, PI, and PF were achieved by 43%, 53%, and 30% of the patients respectively, which is indicative of ceiling effects and forces the distributions into (non-normal) asymmetric, tailed types. The ceiling effect was consistently more apparent in men, i.e., 47% versus 19% in women for PF. The best possible OKS scores were achieved by 8% of the patients.

The GTO showed moderate to large correlation with the short form change scores: r_s_ confidence intervals for PAIN and PI were located above 0.3 with a negligible overlap with the preset threshold of 0.3 for PF (0.29; 0.58). The percentage of improved patients was 92%. The results for the three MIC approaches are shown in Table 4.

**Table 4:** Results for different calculations of Minimal Important Change (absolute values)

	Correlation^a^ between baseline and follow-up scores	MIC_ROC_^b^	MIC_pred_^b^	MIC_adj_^b^
PAIN	0.20 (*p* = 0.02)	10.01 (5.50 to 17.85)	12.92 (10.48 to 15.38)	7.15 (3.67 to 10.92)
PI	0.38 (*p* = 0.00)	8.31 (4.90 to 11.35)	8.76 (6.22 to 10.88)	3.53 (0.09 to 6.61)
PF	0.56 (*p* = 0.00)	8.38 (5.75 to 9.35)	8.12 (7.10 to 9.14)	5.65 (4.26 to 7.13)

*MIC*_*ROC*_ minimal important change determined with the receiver operating characteristic (ROC) curve; *MIC*_*pred*_ predicted MIC; *MIC*_*adj*_ adjusted predicted MIC; *PAIN* pain intensity; *PI* pain interference; *PF* physical function

^a^Pearson correlation coefficient, r

^b^Results are given as the mean MIC and 95% confidence interval after bootstrapping (n = 1000)

## Discussion

In this longitudinal study with patients receiving TKA, we investigated the measurement properties of three PROMIS short forms by using the condition-specific OKS as the reference instrument.

Construct validity was confirmed for all three PROMIS short forms, but PAIN showed lower correlations than the other two scales. This might be explained by the factor structure of the OKS. It was confirmed as a unidimensional scale representing a higher-order combined construct of pain and function [38] and it can also be seen as a two-dimensional scale representing pain and function [39]. There are, however, fewer items loading predominantly on pain than on function that might explain the overall lower correlations of OKS with PAIN, while the correlation confidence intervals of OKS with PI and PF were comfortably above 0.5.

We identified good internal consistency and test–retest reliability for the 3- to 4-item PROMIS short forms for pain and function. This is in line with results from Deyo et al. who investigated the measurement properties of the 4-item PF and PI short forms within the PROMIS-29 profile in adults with chronic musculoskeletal pain [40]. They reported Cronbach’s alpha values of 0.92 for PI and 0.86 for PF, but considerably lower ICC confidence intervals compared to those reported in our study. This deviation can be explained by the longer test–retest interval of 3 months versus our shorter period of ≤ 2 weeks, where subjects are considered more stable. Our test–retest calculations including all subsequent results (ICC, SEM_agr_, SDC90) could hypothetically be influenced by the number of patients answering with the best possible score on both occasions, test and retest (18% to 20%). Therefore, we conducted an a posteriori analysis with results shown in Additional file 1: Table 5. This analysis showed that the ICC was indeed inflated up to a difference of 0.1. When the ICCs are calculated without these subjects, the values are lower but still acceptable (≥ 0.8).

Responsiveness was acceptable for PAIN and PI, but limited for PF. Specifically, the confidence intervals of correlation with the OKS change scores were overlapping with 0.5. We required the whole confidence interval to be located above 0.5, which is rather conservative. Nevertheless, the observed overlap indicates that our data are compatible with correlations below the preset threshold, and this may be due to the precision level of the estimates based on the sample size we used, especially for the male subsample. It might also be due to the ceiling effects found in the PROMIS short forms affecting the distribution of change scores (see Fig. 2). We re-calculated a posteriori the correlation of change scores without ceiling cases, but still found the lower limit of the correlation confidence intervals below 0.5. The number of items, especially in the PF short form might be too small to show responsiveness at the methodologically required level. From a randomised controlled trial evaluating the effects of 12-week tai chi and physical therapy on patients with symptomatic knee osteoarthritis, authors reported high responsiveness of the 10-item short form for PF and moderate responsiveness of the 6-item short form for PI with ceiling effects (best possible score) for the latter [8].

There is a knowledge gap on MIC thresholds for the investigated PROMIS scales in knee arthroplasty patients. In addition, there is still a wide variety of terminologies and calculation methods used for the concept(s) of MIC and its estimation. We agree with the recent recommendation advocating anchor-based over distribution-based methods for determining meaningful change estimates [41]. In recent years, there has been constant development of the anchor-based MIC calculation to account for bias as the disproportional size of improved versus non-improved patient groups and reliability of the transition rating [31–33, 41]. On adopting this approach, our values of MIC_adj_ ranged from 3.5 to 7.15 and were smaller than the MICs derived from other calculation methods (8.12 to 12.9). Of note, the large confidence intervals of MIC_adj_ for PI and PAIN indicate that our point estimates may be imprecise. Our MIC estimates are partly in line with reported values from others. For example, Hung et al. reported the MIC_ROC_ for PROMIS PF CAT as a T-score change of 8 for an orthopaedic patient population with hip and knee joint disorders (68% improved patients) [42]. According to the analysis of Terluin and colleagues, the MIC_ROC_ is overestimated when the proportion of improved patients is larger than 0.5 [31]. We determined an SDC90 of about 8 points, which means that if the true (genuine) MIC is smaller than 8, it cannot be distinguished from measurement error on an individual level. Two further studies suggested MIC values of 2 to 3 points for PI considering various MIC calculation methods in a mixed sample of patients with either chronic low back pain or hip or knee osteoarthritis pain [43] and estimated from the mean score change in the validation study on the PROMIS-29 profile [40]. Most likely this cannot be detected on an individual level due to our estimated SDC90 of about 8 points.

Compared to the OKS, all short forms show smaller effect sizes based on SEM_agr_, which means that the joint-specific OKS allows a more detailed grading of patient recovery than the generic PROMIS short forms for pain and function.

For the follow-up of knee arthroplasty patients, the high proportion of patients with best possible scores for PI and PAIN after surgery may not be critical. These scales represent unipolar constructs where the absence of pain can no longer be differentiated. However, the ceiling effect for PF is problematic. Researchers should be careful in interpreting PF short form score changes. If the maximum score is reached by an individual, their current functional state might be underestimated, and further improvement cannot be measured. This problem may be resolved by using PF CAT without substantially increasing respondent burden. The OKS, which incorporates both pain and function, did not show such a ceiling effect.

### Limitations

Only 67% of eligible patients responded at baseline and 56% at follow-up. We excluded the largest group of patients who are, in fact, currently being followed up for a condition affecting their other knee, and this was done to decrease response burden. The second largest group of excluded patients, who were either living abroad or did not speak German, lack the most basic characteristic essential for validating German language questionnaires and had to be excluded to ensure study population representativeness. From our internal registry quality control procedures, we know that “lack of time” is the most common reason for not responding and less than 3% refused to cooperate because they were dissatisfied with their treatment, which suggests that this study was not prone to a major source of selection bias.

Because our sample comprised primarily participants from German speaking Switzerland, this aspect might nonetheless limit the generalisability of our results. Our OKS baseline score is comparable to that reported for two British TKA cohorts from the period 2010 to 2016 (n = 575) [44], but is higher (indicative of less knee pain and better function) than that reported for the 2009 to 2011 National Health System data set (n = 101,036) [45] and a British multicentre study spanning 2013 to 2016 (n = 709) [17].

Regarding the GTO, we are aware that the external criterion measure we used might not be ideal in terms of recommendations given for transition ratings, for example, the use of balanced 7- to 11-point numerical scales with written descriptors on the ends and at the midpoint [35]. We acknowledge that the choice of a global versus domain-specific transition questions can influence the results of the MIC. The global character of this question allows the patient to consider other constructs than only pain and function for the evaluation of their clinical situation. Above all, our analysis of MIC suffered from non-adequate data in terms of distribution and the proportion of improved patients. Further research is needed to determine how to calculate MICs for interventions with generally large effects and rare failure rates such as TKA.

The ceiling effects observed in the test–retest samples led to a slight underestimation of SEM_agr_ and SDC90. Results of the analysis without ceiling cases can be found in the Additional file 1: Table 5.

## Conclusion

Reasons for using PROMIS short forms may be that one wants to use a generic measure to compare different patient groups while the possibility for using CAT technology is missing. In our study using the shortest available short forms, we showed that this strong reduction to 3 to 4 items comes at the expense of responsiveness and that one loses measurement accuracy in patients with good recovery. This fact needs to be strongly considered within study planning. Responsiveness determines the power of a study and good responsiveness is the key to detect differences between treatments [46].

While we could provide a lot of valuable information about measurement properties of the PROMIS short forms for pain and function using data from our routine clinical registry, there is still some uncertainty about MIC thresholds since the confidence intervals around our point estimates are large.

## Supplementary Information

**Additional file 1**. **Supplement Table 5**. Reliability, agreement and smallest detectable change calculated from test-retest sample excluding patients presenting the best possible PROMIS short form scores on both test occasions.

## Data Availability

The datasets used and/or analysed during the current study are available from the corresponding author on reasonable request.

## Abbreviations

CAT: Computer adaptive testing
COSMIN: COnsensus-based Standards for the selection of health status Measurement INstruments
GTO: Global treatment outcome
ICC: Intraclass correlation coefficient
MIC: Minimal important change
OKS: Oxford Knee Score
PAIN: Pain intensity
PF: Physical function
PI: Pain interference
PROMIS: Patient Reported Outcomes Measurement Information System
ROC: Receiver operating characteristic
SDC (SDC90): Smallest detectable change for individuals (that can be considered above the measurement error with a 90% confidence level)
SEM_agr_: Agreement assessed using the standard error of measurement
