# Correction: Measurement properties of PROMIS short forms for pain and function in patients receiving knee arthroplasty

**DOI:** 10.1186/s41687-023-00590-y

**Published:** 2023-05-26

**Authors:** Anika Stephan, Vincent A. Stadelmann, Stefan Preiss, Franco M. Impellizzeri

**Affiliations:** 1 Department of Teaching, Research and Development – Lower Extremities, Schulthess Clinic, Lengghalde 2, 8008 Zurich, Switzerland; 2 Knee Surgery, Schulthess Clinic, Lengghalde 2, 8008 Zurich, Switzerland; 3 Faculty of Health, University of Technology Sydney, PO Box 123, Broadway, NSW 2007 Australia

**Correction: Journal of Patient-Reported Outcomes (2023) 7:18** 10.1186/s41687-023-00559-x

Following publication of the original article [1], the authors identified that the original colours did not display correctly in Table 2. The correct table is given below.

**Table 2:**
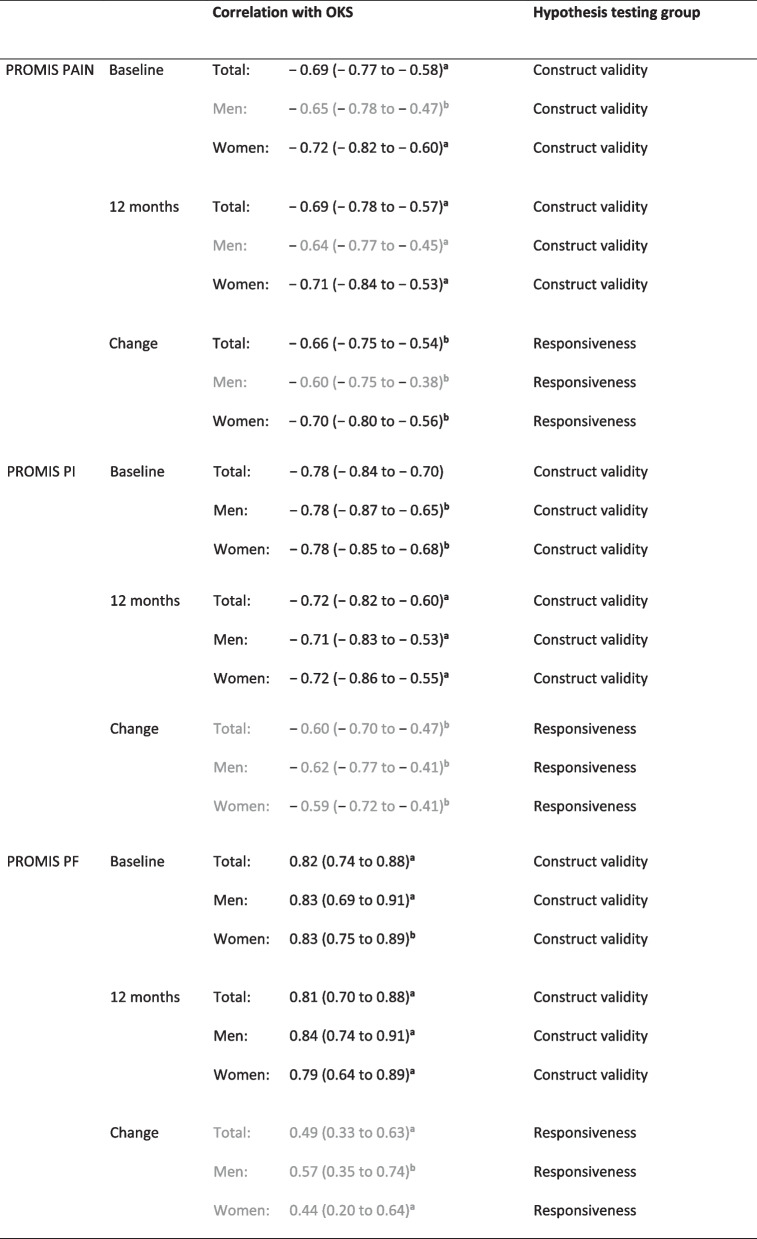
Correlations between PROMIS scales and the OKS

*PROMIS* Patient Reported Outcomes Measurement Information System; *OKS* Oxford Knee Score; *PAIN* pain intensity; *PI* pain interference; *PF* physical function; black versus grey font color: the confidence interval of the correlation does not overlap/overlaps with the preset correlation threshold

^a^Spearman's rank correlation coefficient, r_s_

^b^Pearson’s correlation coefficient, r

The original article [1] has been updated.
