# Supplementary material for: Measurement properties of PROMIS short forms for pain and function in patients receiving knee arthroplasty
Source: J Patient Rep Outcomes. 2023 Feb 28;7:18. doi: 10.1186/s41687-023-00559-x (PMC9975126; doi:10.1186/s41687-023-00559-x)
Supplement: Supplementary file 1 — Additional file 1. Supplement Table 5. Reliability, agreement and smallest detectable change calculated from test-retest sample excluding patients presenting the best possible PROMIS short form scores on both test occasions. [file 41687_2023_559_MOESM1_ESM.docx]

**Supplement**

Table 5 Reliability, agreement and smallest detectable change calculated from test-retest sample excluding patients presenting the best possible PROMIS short form scores on both test occasions

|  |  | **n** | | **ICC**^a^ | **SEM_agr_** | **SDC90** | **Effect size based on SEM_agr_** |
| --- | --- | --- | --- | --- | --- | --- | --- |
| PROMIS PAIN |  | 39 | | 0.86 (0.75 to 0.92) | 4.02 | 9.38 | 4.86 |
|  | | |  | | | | |
| PROMIS PI |  | 40 | | 0.80 (0.66 to 0.89) | 3.73 | 8.70 | 4.33 |
|  | | |  | | | | |
| PROMIS PF |  | 41 | | 0.93 (0.87 to 0.96) | 1.90 | 4.44 | 5.73 |

ICC, intraclass correlation coefficient; SEMagr, agreement assessed using standard error of measurement; SDC90, smallest detectable change for individuals that can be considered above the measurement error with a 90% confidence level; Effect size based on SEM_agr_, calculated as absolute value of the mean change score divided by SEM_agr_; PROMIS, Patient Reported Outcomes Measurement Information System; PAIN, pain intensity; PI, pain interference; PF, physical function

^a^ 95% confidence interval in parentheses
